# Spatial Localization and Binding of the Probiotic *Lactobacillus farciminis* to the Rat Intestinal Mucosa: Influence of Chronic Stress

**DOI:** 10.1371/journal.pone.0136048

**Published:** 2015-09-14

**Authors:** Stéphanie Da Silva, Catherine Robbe-Masselot, Arthur Raymond, Myriam Mercade-Loubière, Christel Salvador-Cartier, Bélinda Ringot, Renaud Léonard, Isabelle Fourquaux, Afifa Ait-Belgnaoui, Pascal Loubière, Vassilia Théodorou, Muriel Mercier-Bonin

**Affiliations:** 1 Université de Toulouse, INSA, UPS, INP, LISBP, 135 Avenue de Rangueil, F-31077 Toulouse, France; 2 INRA, UMR792 Ingénierie des Systèmes Biologiques et des Procédés, F-31400 Toulouse, France; 3 CNRS, UMR5504, F-31400 Toulouse, France; 4 UMR 1331 TOXALIM INRA/INPT/UPS, Equipe de NeuroGastroentérologie et Nutrition, 180 chemin de Tournefeuille, 31027 Toulouse cedex 9, France; 5 Univ Lille Nord de France, USTL, Unité de Glycobiologie Structurale et Fonctionnelle, IFR 147, F-59650 Villeneuve d'Ascq, France; 6 CNRS, UMR 8576, F-59650 Villeneuve d'Ascq, France; 7 Faculté de Médecine Rangueil, Centre de Microscopie Electronique Appliquée à la Biologie (CMEAB), Bât. A5, 133 route de Narbonne, 31062 Toulouse cedex, France; 8 Lallemand SA, 19 Rue des Briquetiers, 31702 Blagnac, France; University of Louisville School of Medicine, UNITED STATES

## Abstract

The present study aimed at detecting the exogenously applied probiotic *Lactobacillus farciminis* in rats, after exposure to IBS-like chronic stress, based on 4-day Water Avoidance Stress (WAS). The presence of *L*. *farciminis* in both ileal and colonic mucosal tissues was demonstrated by FISH and qPCR, with ileum as the preferential niche, as for the SFB population. A different spatial distribution of the probiotic was observed: in the ileum, bacteria were organized in micro-colonies more or less close to the epithelium whereas, in the colon, they were mainly visualized far away from the epithelium. When rats were submitted to WAS, the *L*. *farciminis* population substantially decreased in both intestinal regions, due to a stress-induced increase in colonic motility and defecation, rather than a modification of bacterial binding to the intestinal mucin Muc2.

## Introduction

The human intestine is colonized with a complex microbial community, known as the microbiota, which reaches about 10^14^ bacteria and consists of at least 1000 species. This microbiota plays a key role in gut physiology and host health by fulfilling a great number of functions, such as digestion of otherwise unprocessed dietary nutrients, synthesis of vitamins and short-chain fatty acids, modulation of the immune system and inhibition of pathogen colonization [1]. Bacteria may be involved in the pathogenesis and pathophysiology of numerous intestinal diseases, including irritable bowel syndrome (IBS) [2–7].

Probiotic strategies for maintaining or restoring host health through the modulation of intestinal microbiota have gained interest during the past few years [8]. In particular, the efficacy of probiotics in IBS management has been assessed [9], even though the magnitude of benefit and the most effective species and strains remain uncertain [10]. Probiotics are “live microorganisms that, when administered in adequate amounts, confer a health benefit on the host”, as recently proposed by an expert panel, convened by the International Scientific Association for Probiotics and Prebiotics (ISAPP) [11], on the basis of the FAO/WHO definition (FAO/WHO, 2001). Probiotic bacteria are thought to contribute to health through several mechanisms, including competitive exclusion of pathogens, strengthening of the intestinal epithelial barrier and modulation of the host immune system [12–13]. The mucus-binding capacity has been shown to be important for prolonged intestinal residence time [14]. For instance, *Lactobacillus rhamnosus* GG (LGG) expresses cell surface pili to gain this mucus-binding ability [15–16]. Other cell surface proteins may act as mediators of bacterial adhesion to mucus [17–23].

For obvious reasons of sample accessibility, the *in vivo* colonization capacity of probiotics, even transient, has mainly been investigated in fecal or cecal contents [24–25]. However, such data do not provide any information on the spatial localization and distribution of probiotic bacterial cells in the different gut regions, as probed for instance with FISH and strain-specific molecular probes. Valeur *et al*. [26] provided direct evidence of colonization of the stomach, duodenum and ileum by *Lactobacillus reuteri* on biopsy specimens from healthy humans. Moreover, Wang *et al*. [27] showed that *Lactobacillus plantarum* L2, chosen for its *in vitro* adhesive and immuno-modulatory properties, is able to colonize the rat gastrointestinal tract with strong adherence to the ileum and colon and also to the duodenum and jejunum, albeit at a lesser extent. In a further study, Lebeer *et al*. [28] investigated the spatial organization of endogenous lactobacilli and exogenously applied LGG at specific locations in human, murine and avian gastrointestinal tracts. However, all these findings have been reported for healthy humans or animals and the consequences of pathophysiological conditions, like in IBS, on gut colonization by probiotics remain poorly understood.

Based on this background, the present study aimed at detecting the presence of the probiotic *Lactobacillus farciminis* in the rat gut, after exposure to a chronic psychological stress reproducing hallmark features of IBS, such as increased visceral hypersensitivity to colorectal distension and increased gut permeability [29–30], based on 4-day Water Avoidance Stress (WAS) [31]. To this aim, FISH, scanning electron microscopy and quantitative PCR (qPCR) were combined. Special attention was paid to the binding properties of *L*. *farciminis* to the intestinal mucosa and especially to Muc2, the major secreted mucin in the ileum and colon.

## Materials and Methods

### Animals and bacterial cells

9-week old male Wistar rats (Janvier SA), weighing 150–175 g and individually housed in standard polypropylene cages in a temperature-controlled room (22±1°C), were used. Animals were allowed free access to water and food (standard pellets SAFE). *L*. *farciminis* (CIP 103136, Institut Pasteur Collection) was obtained freeze-dried (Lallemand SA) and stored at -20°C. 1-mL probiotic suspension, prepared daily by diluting freeze-dried bacteria in sterile saline (0.9% NaCl (w/v)), was administered by gastric gavage.

### Experimental design

Rats received oral administration of *L*. *farciminis* (10^11^ CFU/day/rat) or vehicle (0.9% NaCl (w/v)) for 14 days. At day 10, they were submitted between 8:00 and 12:00 am (i.e., no effect of circadian rhythm) either to sham stress or WAS for 4 days. Corresponding groups (4 groups, n = 8/group) will be designed in the following as control (vehicle/sham stress), WAS (vehicle/stress), LF (probiotic/sham stress) and LF+WAS (probiotic/stress). For the WAS session, rats were placed on a Plexiglas platform (6 x 6 cm^2^) affixed to the center of a transparent plastic container (40 x 60 x 30 cm^3^), filled with room temperature water (25°C) to within 1 cm of the top of the platform, or kept empty (sham stress) for 1 h daily during 4 days. In order to minimize any environmental stress, animals were handled for one week prior to the experiments. After the last sham or WAS session, rats were killed by decapitation and the gastrointestinal tract was aseptically removed to collect ileal and distal colonic sections for further analysis (see below). Toxalim animal facility (INRA, UMR 1331, Toulouse, France) is licensed by the French Ministry of Agriculture (agreement n° B31.555.13). All animal experiments complied with the European Union regulation and were approved by the regional ethics committee Midi-Pyrénées (approval MP/02/60/11/11).

### FISH analysis

16S rRNA-targeted FISH molecular probes, purchased from Eurogentec (Eurogentec S.A., Belgium), were synthesized with a FITC (fluorescein isothiocyanate) or Cy3 (cyanine 3) reactive fluorescent dye at the 5’ end. The Eub338 universal bacterial probe (5’-GCTGCCTCCCGTAGGAGT-3’) was used to detect all relevant bacteria [32]. The SFB-specific oligonucleotide probe (5’-GCGAGCTTCCCTCATTACAAGG-3’) was based on the work of Snel *et al*. [33]. The Lfarc probe (5’-AGCTTCAATCTTCAGGAT-3’) was chosen for *L*. *farciminis*. Its specificity was previously evaluated by analysis of hybridization with different LAB genera, including *Lactobacillus*, *Leuconostoc*, *Pediococcus* and *Oenococcus* [34]. No cross-reaction, false negative or unspecific probe binding was found. Intestinal segments were fixed in Carnoy’s solution during 6–12 h at room temperature and embedded in paraffin using standard procedures. Prior to FISH analysis, 5-μm thickness serial paraffin sections were placed on positively charged slides (SuperFrost Plus). These slides were immersed in toluene for deparaffination. Then, samples were rehydrated by transfer to a series of aqueous ethanol solutions with decreasing percentage of ethanol, followed by two 2-min washing steps in Milli-Q grade water. A lysozyme treatment was used (10 mg/mL, 30 min, 37°C) to favor permeabilization of bacterial cell walls, followed by washing with milli-Q grade water. Subsequently, 20 μL of hybridization buffer (180 μL NaCl 5 M, 20 μL Tris—HCl 1 M pH 8, 1 μL SDS 20%, 800 μL Milli-Q grade water) and 2 μL of the required FISH probe (50 ng/μL) were spotted onto the sample. To prevent any cross-over between the different probe solutions, each tissue section was circled with a PAP pen (Electron Microscopy Sciences). Hybridization was performed during 2 h at 45°C in a humid chamber (Grant Boekel). After hybridization, slides were washed with 50 μL of buffer (900 μL NaCl 5 M, 100 μL Tris–HCl 1 M pH 8, 2.5 μL SDS 20%, 4 mL Milli-Q grade water) for 10 min at 48°C, rinsed with Milli-Q grade water and air-dried. Slides were then mounted with Antifade-containing DAPI (Invitrogen) to counterstain cell nuclei, and examined by epifluorescence microscopy. Images were processed using Leica FW 4000 view software (Leica).

### Scanning electron microscopy

Tissues were fixed in 2% glutaraldehyde in 0.1 M Sorensen phosphate buffer (pH 7.4) for 1 h at 4°C, washed 3 times during 10 min in deionised water. Samples were then dehydrated in a series of graded ethanol solutions, dried by critical point drying with Leica EMSCOPE CPD 750, coated with gold-palladium for 5 min at 100 Å/min, and observed with a S450 scanning electron microscope (Hitachi), at an accelerating voltage of 15 kV.

### DNA extraction

DNA from ileal and colonic samples was extracted. Briefly, tissues were disrupted with 2 cycles (6.5 M/s, 30 s) of Fast Prep (MP Bio) in Lysing Matrix A tubes (MP Bio), followed by 2 cycles (6.5 M/s, 30 s) of Fast Prep (MP Bio) with 0.6 g of glass beads (Sigma). DNA was extracted and purified using a Wizard Genomic DNA Purification kit (Promega) following the manufacturer’s instructions. An enzymatic step was added with lysozyme (20 mg/mL) and mutanolysin (10 U/μL) for 1 h at 37°C. DNA concentration was subsequently determined by NanoDrop ND-2000 (NanoDrop) and DNA quality was checked by electrophoresis.

### Real-time qPCR analysis of bacterial 16S rRNA gene

The population of total bacteria, lactobacilli and *L*. *farciminis* in mucosal tissues for each group of rats was evaluated by qPCR analysis targeting bacterial group-specific 16S rRNA. The total bacterial population was amplified with universal primers, Ubac_for (5'-TCCTACGGGAGGCAGCAGT-3') and Ubac_rev (5'-GGACTACCAGGGTATCTAATCCTGTT-3') [35]. Lactobacilli, and more particularly *L*. *farciminis*, were quantified using specific primers, namely LaB_F362 (5'-AGCAGTAGGGAATCTTCCA-3') and LaB_R677 (5'-CACCGCTACACATGGAG-3') [36], and Farci_for (5'-GCCGCAAGGAATTTATCTTCAA-3') and Farci_rev (5'-TCCCCCGCCACCTGTAG-3'), respectively.

Amplification was carried out in a final volume of 25 μL containing 12.5 μL of SYBR Green Supermix (BioRad), 5 μL of DNA templates, 2 μL of each primer (10 μM) and 3.5 μL of Milli-Q grade water. Reactions were performed in a MyIQ Single Color cycler (BioRad). Thermocycling conditions were the following: initial DNA denaturation at 95°C for 3 min, 40 cycles of denaturation at 95°C for 15 s, primer annealing at 60°C for 45 s with fluorescence detection. Following amplification, melting curves were determined by 70 cycles beginning at 60°C with a stepwise increase in temperature (0.5°C each 10-s period) until 95°C. Standard curves generated from 10-fold serial dilutions of DNA samples of given strains were used for quantification of total bacteria, lactobacilli and *L*. *farciminis*. Intestinal samples were analyzed for each group of rats by qPCR in duplicate. Using cycle threshold values in the linear range, bacterial equivalents were deduced from the standard curves. Results for *L*. *farciminis* are expressed as the percentage of *L*. *farciminis* to the total lactobacilli.

### Assay of *L*. *farciminis* adhesion to the intestinal mucosa

*L*. *farciminis* was labeled with fluorescein isothiocyanate (FITC, Sigma Aldrich) and adhesion assays were performed as previously described [31]. Briefly, bacterial cells were resuspended in 0.15 M NaCl/0.1 M sodium carbonate pH 9 and incubated for 1 h in 10 mg/mL FITC in DMSO (DMSO, Sigma Aldrich). After centrifugation, the bacterial suspension was 5-fold diluted in blocking buffer (Protein free blocking buffer, Thermo Fischer Scientific Inc, USA). Sections of 5-μm thickness were then prepared from paraffin block of Carnoy-fixed ileal and colonic tissues for control and WAS conditions. After deparaffination, rehydration and saturation of tissue sections, bacterial cells were incubated for 2 h at room temperature. In parallel, mucin immunohistochemical staining was realized by using polyclonal primary antibody against MUC2 (H300 sc-15334, Santa Cruz Biotechnology, diluted 1:100). Sections were washed with PBS before incubation with secondary antibody coupled to ALEXA Fluor 546 (Invitrogen, USA, diluted 1:250). Slides were counterstained with DAPI and mounted using 1% DABCO mounting medium (80% glycerol), sealed and dried overnight before examination.

### 1-D bacterial overlay

The 1-D bacterial overlay procedure was adapted from Odenbreit *et al*. [37] to evaluate the binding of *L*. *farciminis* to Muc2. In brief, secreted Muc2 was purified from scrapped ileal and colonic mucus, for control and WAS conditions, as previously described [31]. Muc2 (10 μg) was spotted on dry nitrocellulose membranes. BSA was used as negative control. Bacteria (10^9^ CFU/mL in phosphate-buffered saline) were labeled with DAPI for 15 min at room temperature in the dark. Labeled bacteria were added to the membrane in blocking buffer. After incubation during 1 h at room temperature in the dark, the fluorescence of adherent bacteria was detected by a ChemiGenius 2 imaging system (Syngene).

### Statistical analyses

For qPCR standard curves, linear regression significance was analyzed with Pearson’s test. Data are reported as means ± SEM (n = 8). Two-way analysis of variance (ANOVA), followed by Bonferroni’s post-test, was performed for grouped columns. Significance was set at *p-value*<0.05 (*) or *p-value*<0.001 (***). All tests were performed with GraphPad Prism 4.00 (GraphPad Software Inc.).

## Results

### Spatial localization and cell morphology of total bacteria within the ileum and colon

**Fig 1:**
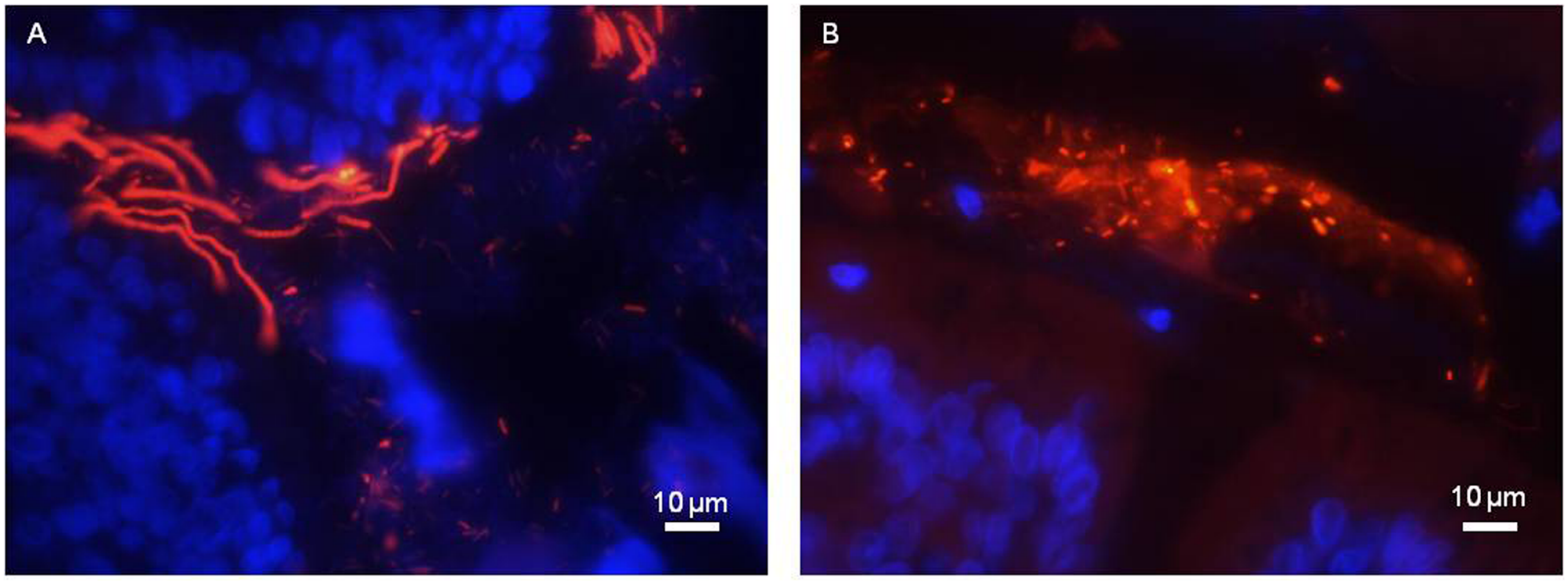
FISH for spatial organization and cell morphology of total bacteria in the rat gastrointestinal tract. The example of *L*. *farciminis*-fed and sham-stressed rats (LF group) is given. Ileal (A) and colonic (B) mucosal tissues were analyzed by FISH using a Eub338 universal probe. Bacteria are visualized in red and cell nuclei in blue with DAPI staining (scale bar 10 μm). In the ileum, bacterial cells were observed as freely dispersed; when located close or in direct contact with the epithelium, they mainly exhibited a long filamentous and segmented shape. In the colon, spatial organization and cell morphology were different: bacteria were localized far away from the epithelium, either as dispersed and, occasionally, rod-like shaped cells or as micro-colonies.

**Fig 2:**
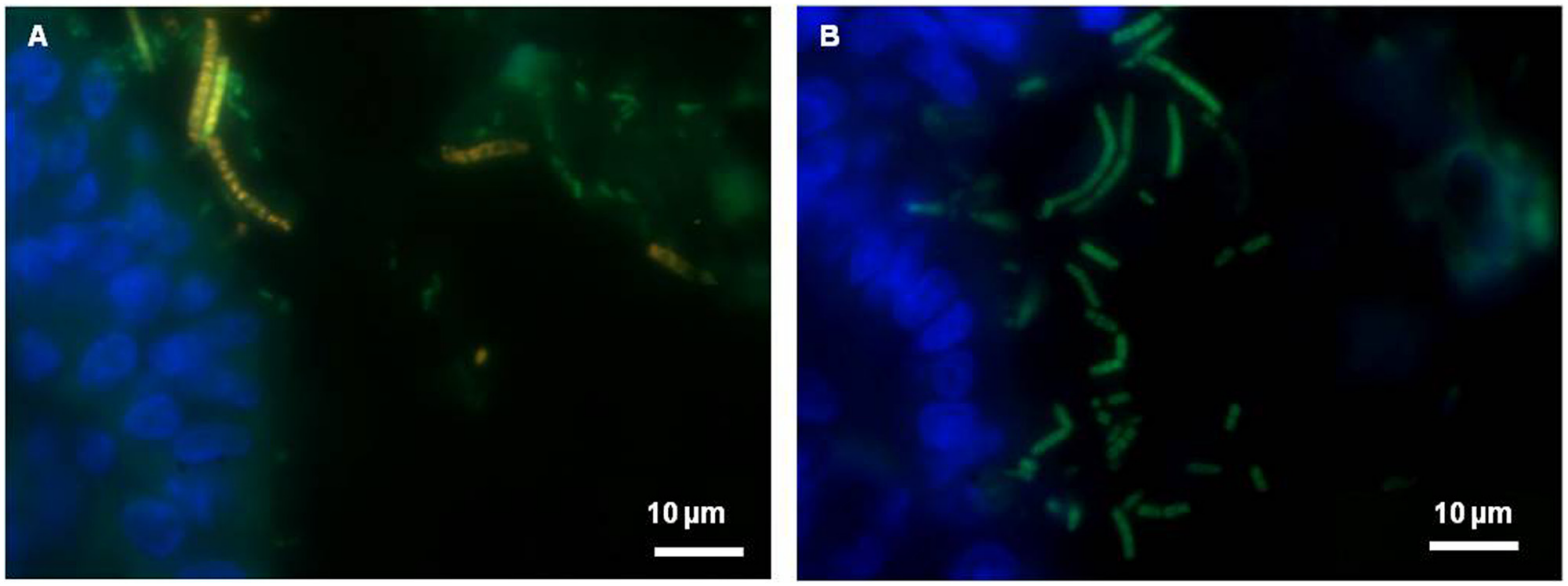
FISH for spatial organization and cell morphology of SFB within the rat ileum. The example of *L*. *farciminis*-fed and sham-stressed rats (LF group) is given. (A) Ileal mucosal sample co-stained with a SFB-specific probe labeled with Cy3 (in red) and with an universal Eub338 probe labeled with FITC (in green). SFB are visualized in yellow/orange; (B) Ileal mucosal sample stained with a SFB-specific probe labeled with FITC. SFB are visualized in green. Cell nuclei are detected in blue with DAPI staining (scale bar 10 μm). The filamentous and segmented cell morphology of SFB was easily recognized, with bacterial cells closely approaching and even anchoring to the epithelium.

**Fig 3:**
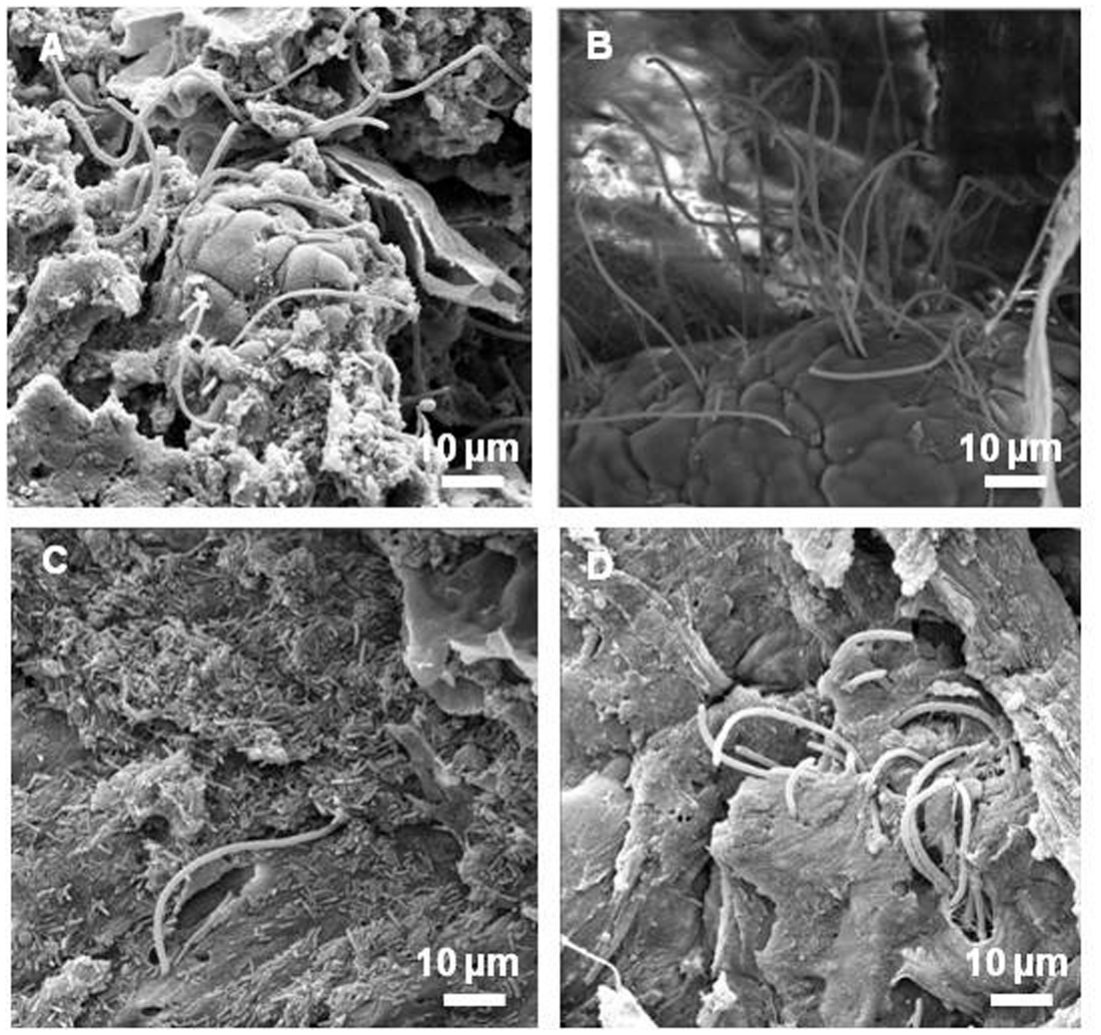
Scanning electron microscopy for spatial organization and cell morphology of SFB within the rat ileum. (A) vehicle-fed and sham-stressed animals (control group), (B) vehicle-fed and stressed animals (WAS group), (C) *L*. *farciminis*-fed and sham-stressed animals (LF group) and (D) *L*. *farciminis*-fed and stressed animals (LF+WAS group) (scale bar 10 μm). Independently of the group under study, SFB were observed, consisting in typical thick filaments exhibiting plump, rounded and well-defined segments with distinct septa and a thin tapered structure at the site of attachment to the epithelium. Note that, for sham-stressed animals fed with the probiotic, a huge amount of rod-shaped *L*. *farciminis*-like bacteria was observed and after stress, this population was dramatically decreased.

FISH with a universal probe was used to visualize the spatial organization of bacterial communities in mucosal tissues. DAPI staining allowed eukaryotic cell nuclei to be observed. An example obtained from *L*. *farciminis*-fed and sham-stressed animals (LF group) is shown in Fig 1 and displays transverse sections of mucosal tissue from the ileum and the colon (Fig 1A and 1B, respectively). In the colon, bacteria were seen to be localized far away from the epithelium with a separation distance of about 30 μm, either as dispersed and, occasionally, rod-like shaped cells or as micro-colonies (Fig 1B). In the ileum, dispersed cells were also observed in the lumen but closer or even in direct contact with the epithelium, bacteria mainly exhibited a typical long filamentous shape with segments (Fig 1A), probably corresponding to Segmented Filamentous Bacteria (SFB). To confirm such hypothesis, further FISH analysis with a SFB-specific probe was carried out. Results are displayed in Fig 2 for the ileum of *L*. *farciminis*-fed and sham-stressed rats (LF group). The characteristic filamentous and segmented cell morphology was easily recognized (Fig 2A), with bacteria closely approaching and even anchoring to the epithelial cells, all along the ileal mucosa (Fig 2B). Consistent with these results, scanning electron microscopy revealed SFB present in the ileum of animals from the LF group (Fig 3C). For the other groups tested, the spatial organization and cell morphology of SFB remained unchanged (Fig 3A, 3B and 3D). Note that no SFB were detected in the colonic mucosa for all conditions under study.

### Spatial localization of *L*. *farciminis* within the ileum and colon

**Fig 4:**
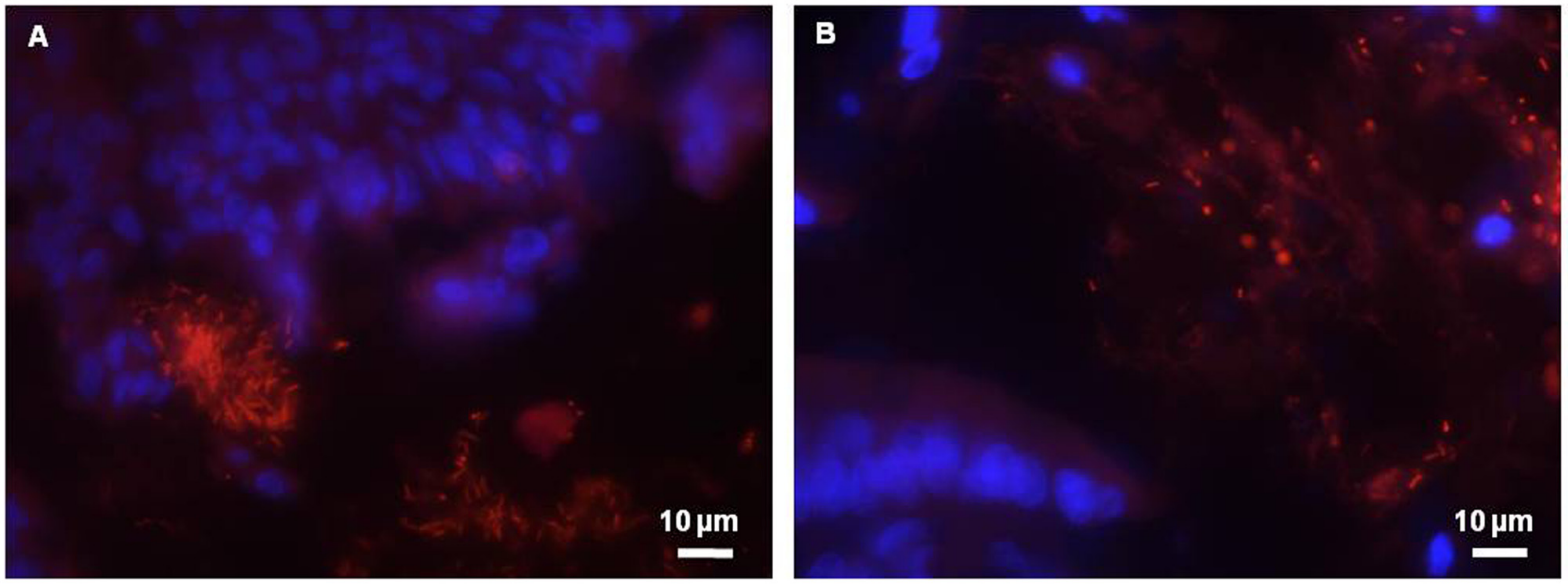
FISH for spatial organization of *L*. *farciminis* in the rat gastrointestinal tract. The example of *L*. *farciminis*-fed and sham-stressed rats (LF group) is given. Ileal (A) and colonic (B) mucosal tissues were analyzed by FISH using a *L*. *farciminis* specific probe. Bacteria are visualized in red and cell nuclei in blue with DAPI staining (scale bar 10 μm). In the ileum, *L*. *farciminis* was detected as micro-colonies more or less close to the epithelium whereas, in the colon, bacteria were mainly visualized far away from the epithelium.

FISH analysis with a specific probe was performed to detect the presence of *L*. *farciminis* within the rat gut. An example obtained from *L*. *farciminis*-fed and sham-stressed animals (LF group) is shown in Fig 4. Images clearly demonstrated the presence of the probiotic in the ileal and colonic mucosal tissues (Fig 4). In the ileum, bacterial cells were organized in micro-colonies more or less close to the epithelium (Fig 4A) whereas, in the colon, they were mainly visualized far away from the epithelium (Fig 4B).

### Quantification of *L*. *farciminis* by qPCR within the ileum and colon

**Fig 5:**
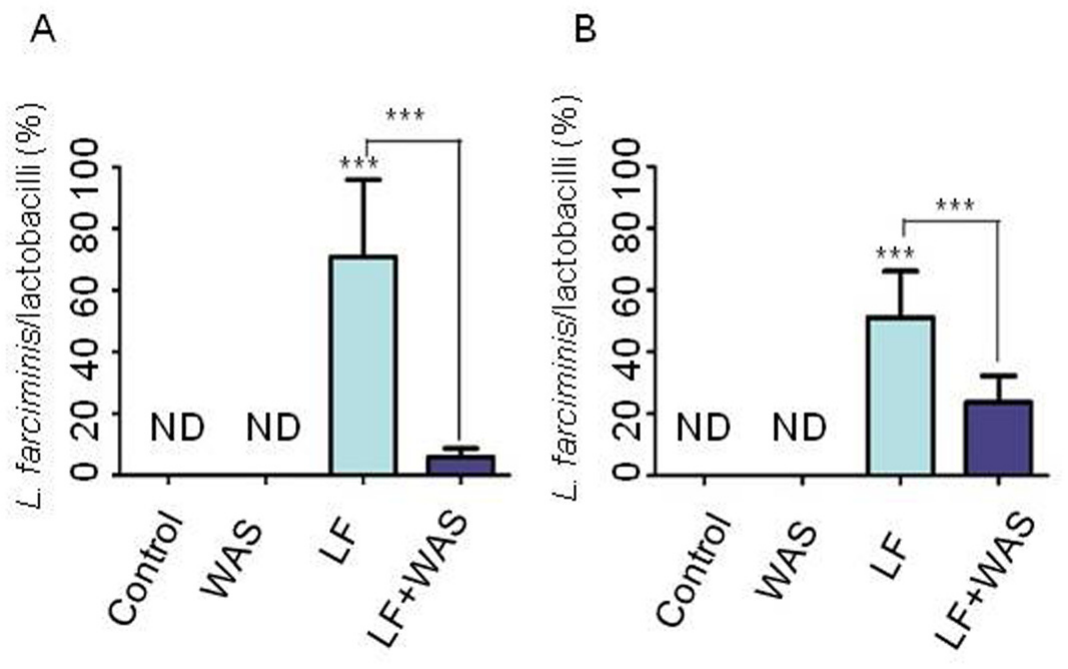
Quantification of *L*. *farciminis* within the rat gastrointestinal tract: influence of stress. Ileal (A) and colonic (B) mucosal tissues were analyzed by qPCR using *L*. *farciminis* and lactobacilli specific probes. The abundance of *L*. *farciminis* was expressed as the ratio of both values. Results given are means ± S.E.M.; n = 8 rats/group (*p-value* < 0.001 vs. controls or vs. LF group in order to determine the effect of WAS on probiotic-fed rats). ND: not detected. Groups shown are control (sham-stressed animals fed with the vehicle), WAS (stressed animals fed with the vehicle), LF (sham-stressed animals fed with the probiotic) and LF+WAS (stressed animals fed with the probiotic). After probiotic feeding, a 4 day-WAS induced a significant decrease in *L*. *farciminis* abundance, both in the ileum and colon, in comparison with sham-stressed rats.

Mucosal tissues from the ileum and colon were analyzed for each group of rats (control, WAS, LF and LF+WAS groups), using qPCR with *L*. *farciminis* and total lactobacilli specific primers. The relative abundance of *L*. *farciminis* was calculated as the ratio of both values. Results are shown in Fig 5. We should note that no change in the total bacteria level was found with qPCR for each region under study. Similarly, the population of lactobacilli, expressed as their proportion relative to the total bacteria, was not statistically different for the four groups tested and approximately reached 50–70% and 20–40% for the ileum and colon, respectively (S1 Fig). In rats which did not receive the probiotic treatment (control and WAS groups), *L*. *farciminis* was not detected, as expected. After the probiotic feeding on sham-stressed rats (LF group), the *L*. *farciminis* proportion sharply increased both in the ileum and colon, albeit at a lesser extent for the latter (Fig 5A and 5B, respectively). However, when probiotic-fed rats were submitted to WAS (LF+WAS group), the population of *L*. *farciminis* substantially decreased in the two intestinal regions under study, with a 7-fold and 2.5-fold decrease in the ileum and colon, respectively (Fig 5A and 5B). Interestingly, consistent with these results, scanning electron microscopy in the ileal region revealed, for sham-stressed animals fed with the probiotic (LF group), a huge amount of rod-shaped bacteria (not seen in the control and WAS groups), undoubtedly corresponding to *L*. *farciminis* (Fig 3C) and after stress (LF+WAS group), this population was dramatically decreased (Fig 3D).

### Binding of *L*. *farciminis* to the ileal and colonic mucosa

**Fig 6:**
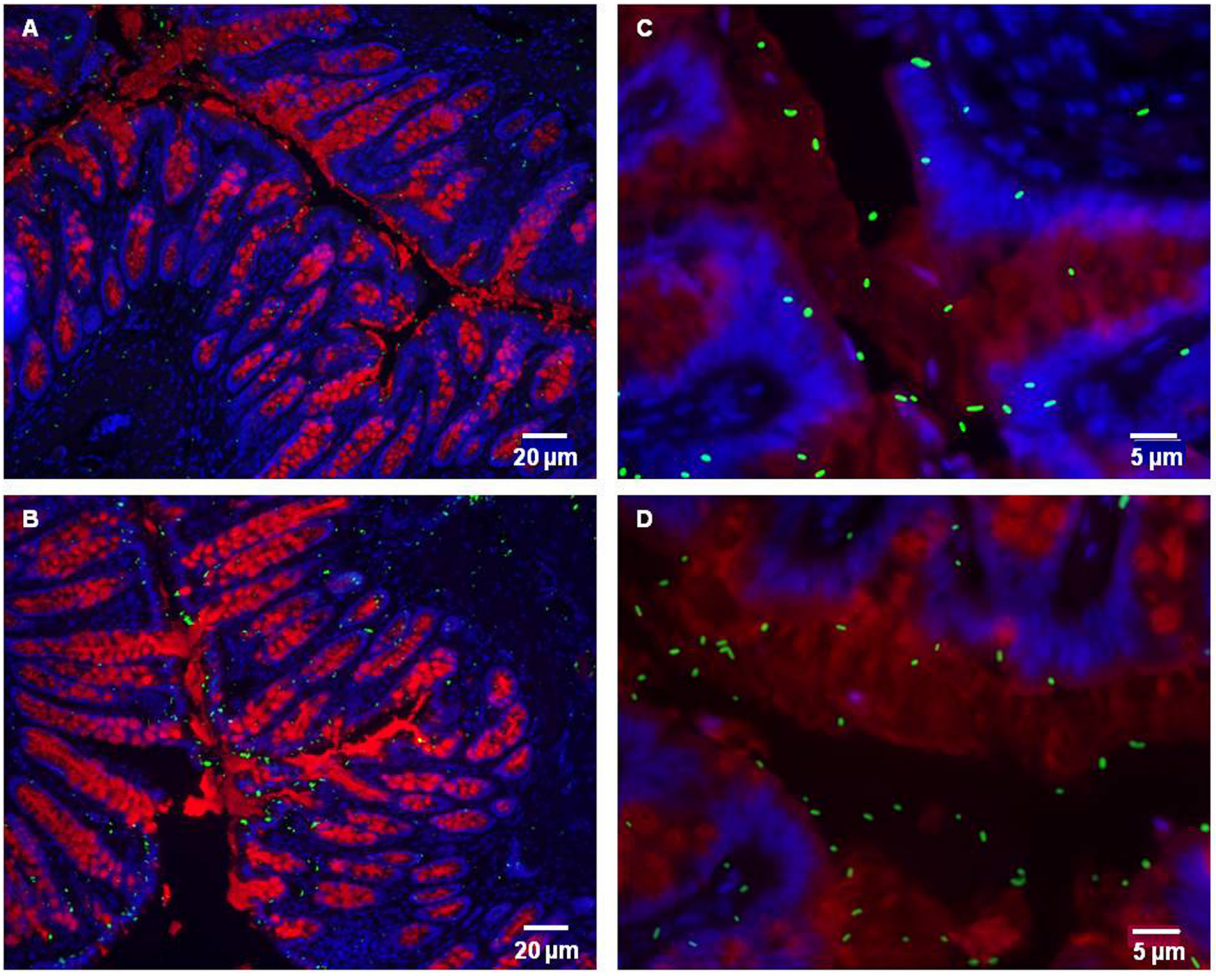
Adhesion of FITC-labeled *L*. *farciminis* to the colonic mucosa. The example of vehicle-fed sham-stressed and stressed rats (control and WAS groups, respectively) is given. (A,C) sham-stressed rats; (B,D) stressed rats. FITC-labeled bacterial cells are seen in green, Muc2 is in red and cell nuclei are in blue (DAPI staining) (A,B: scale bar 20 μm; C,D: scale bar 5 μm). In the colon and independently of stress, *L*. *farciminis* bound to mucus, and notably to Muc2.

**Fig 7:**
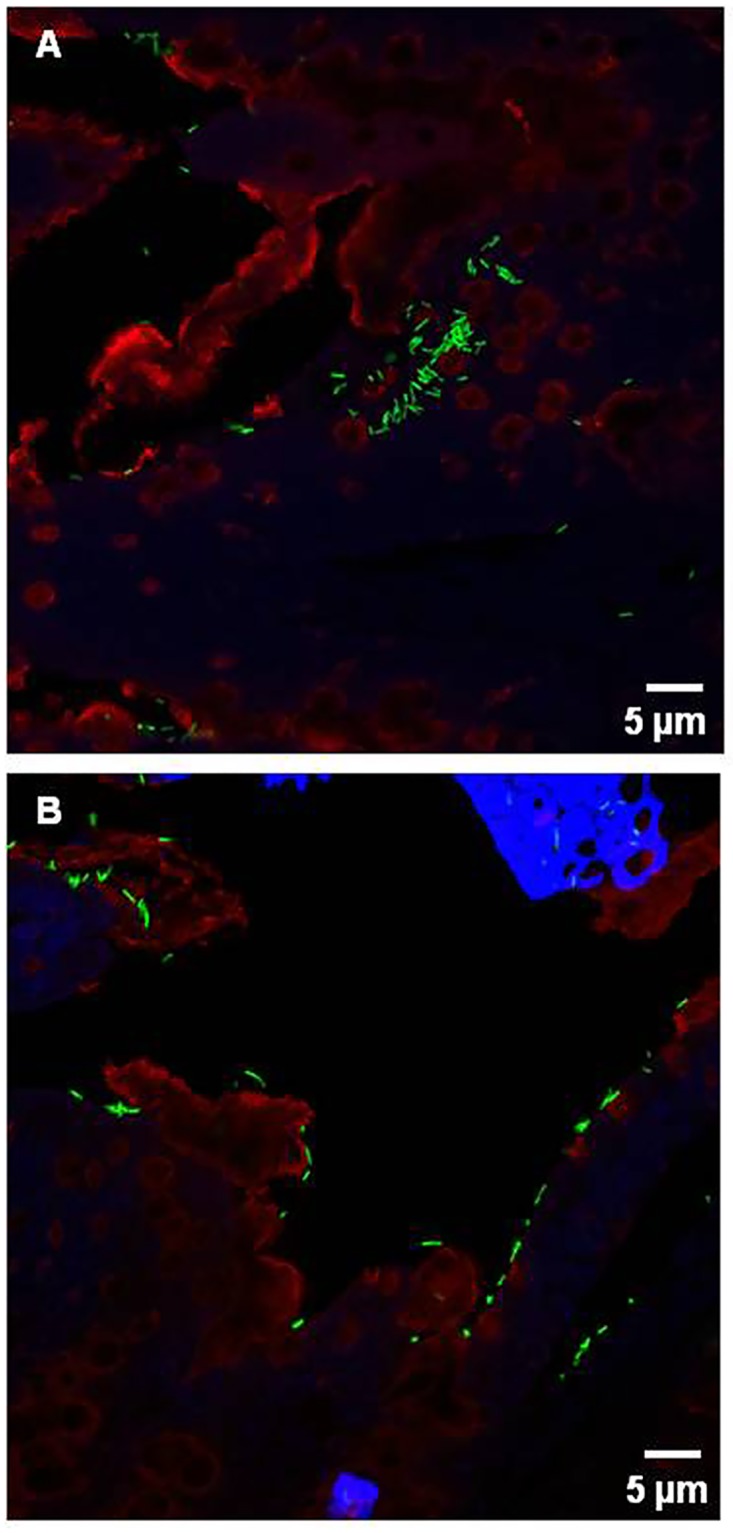
Adhesion of FITC-labeled *L*. *farciminis* to the ileal mucosa. The example of vehicle-fed sham-stressed and stressed rats (control and WAS groups, respectively) is given. (A) sham-stressed rats; (B) stressed rats. FITC-labeled bacterial cells are seen in green, Muc2 is in red and cell nuclei are in blue (DAPI staining) (scale bar 5 μm). In the ileum and independently of stress, *L*. *farciminis* bound to mucus, and notably to Muc2.

The presence of *L*. *farciminis* in the rat gut could be the result of close interactions with the intestinal mucosa and especially the mucus layer. To test this hypothesis, an *ex vivo* binding assay was performed, as previously described [31]: *L*. *farciminis* bacterial cells were labeled with FITC, overlaid on Muc2-stained ileal and colonic tissue sections from vehicle-fed sham-stressed and stressed animals (control and WAS groups, respectively), and then observed using epifluorescence microscopy. In order to avoid any artifact due to *L*. *farciminis* administration, the LF and LF+WAS groups were not considered. Results are shown in Figs 6 and 7 for the control and WAS groups, and for the colon and ileum, respectively. For all conditions, *L*. *farciminis* (in green) bound to mucus (in red), even though a randomly distribution over intestinal lumen and cell nuclei could not be neglected.

### Binding of *L*. *farciminis* to Muc2

**Fig 8:**
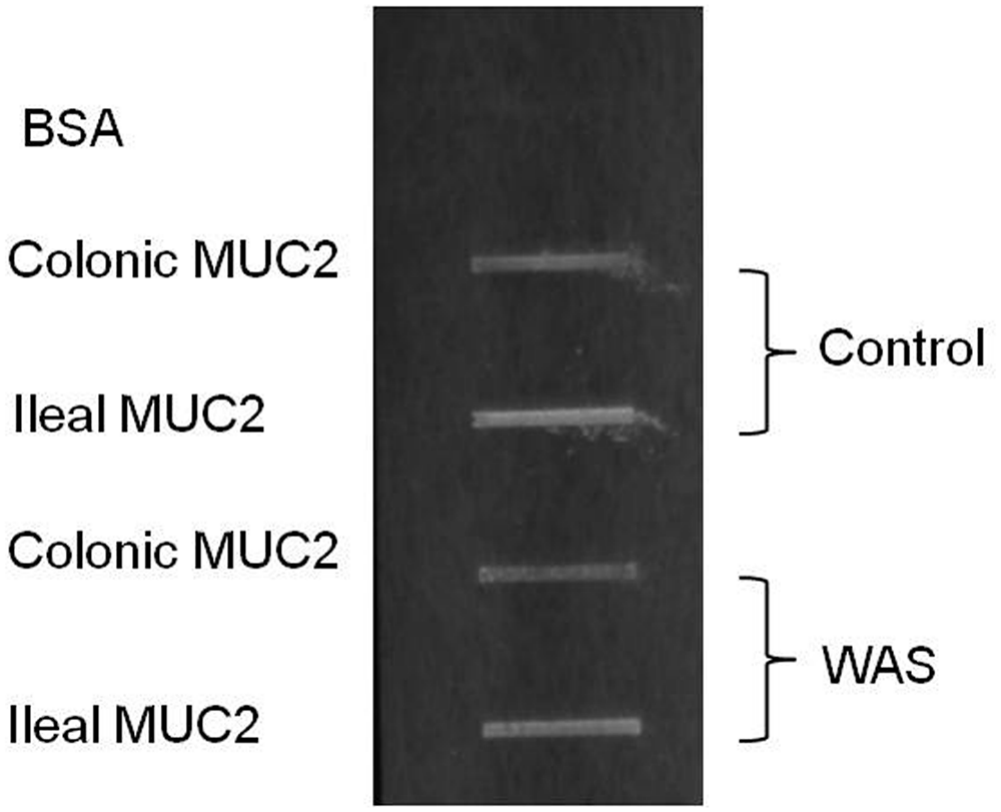
Binding of DAPI-labeled *L*. *farciminis* bacteria to ileal and colonic purified Muc2, explored by slot-blot overlay assays. The example of vehicle-fed sham-stressed and stressed rats (control and WAS groups, respectively) is given. Bacterial binding to BSA is shown as negative control. *L*. *farciminis* tightly bound to ileal and colonic Muc2 in stressed and sham-stressed animals.

In order to refine our understanding of the *L*. *farciminis*/mucus interactions, *in vitro* bacterial binding to Muc2 was assessed on the same groups as those depicted above. To this end, *L*. *farciminis* bacterial cells were labeled with DAPI and overlaid on membranes with immobilized Muc2, purified from mucus of sham-stressed and stressed animals. Bound bacteria were detected by fluorescence. Results are shown in Fig 8 for the colon and ileum. For both regions, *L*. *farciminis* strongly bound to Muc2, as revealed by prominent bands. The binding level for the ileum was slightly higher than that obtained for the colon. Furthermore, no striking differences were observed between control and stressed animals (Fig 8).

## Discussion

The present study aimed at detecting the presence of the probiotic *L*. *farciminis* within the gastrointestinal tract under physiological and pathophysiological IBS-like conditions, using a 4-day WAS model in rats. To this aim, FISH, scanning electron microscopy and qPCR approaches were combined. First, we demonstrated that the population of lactobacilli, revealed by qPCR, was high (50–70% and 20–40% of the total bacteria for the ileum and colon, respectively) and was not statistically different for all conditions under study. In fact, contrary to humans, *Lactobacillus* species represent a significant proportion of the microbiota in rats and can reach 10–15% of the total sequences read [38–40]. Our higher levels were probably due to the different methods used (qPCR vs. clone sequencing methods). In addition, FISH analysis was performed with an universal probe to visualize the spatial organization of the mucosa-associated microbiota. In the colon, consistent with the physical barrier exerted by the inner dense mucus layer [41], bacteria were mainly localized in the lumen and/or the outer mucus layer, either as dispersed cells or as micro-colonies. Such distribution in micro-colonies was previously depicted for healthy mucosal tissues in humans [42–43]. Live/dead staining of these structures showed that most of bacteria were living, particularly those close to the mucosal surface [42]. In our study, in the ileum, and probably due to the "patchy" organization of the mucus layer [41], bacterial cells were also present closer or even in direct contact with the epithelium. Using specific-probe FISH, we assigned these mucosa-attached bacteria to SFB and revealed their characteristic filamentous and segmented morphology, further confirmed by scanning electron microscopy, as depicted in earlier studies [44–45].

Probiotics have widely been used for alleviating IBS symptoms in humans, albeit with sometimes contrasted results [46–50]. In rodents, the ingestion of probiotics was found to improve intestinal barrier function and to protect against visceral hypersensitivity in IBS-like models, based on acute [51–52] or chronic stress [53–54]. Likewise, previous studies of our group showed that *L*. *farciminis* given daily for 15 days was able to reverse partial restraint stress-induced hypersensitivity, increase in colonic paracellular permeability and colonocyte MLC phosphorylation [51]. Such protective effect mainly occurred *via* inhibition of contraction of colonic epithelial cell cytoskeleton and subsequent tight junction opening, and probably involved the direct or indirect role of nitric oxide produced by this probiotic in the lumen [55]. In our recent study [31], *L*. *farciminis* was shown to prevent WAS-induced visceral hypersensitivity, as well as impairment of the mucus and epithelial barriers. To address the origin of this large set of beneficial effects, Lamine *et al*. [56] used culture-based methods for assessing the survival and presence of *L*. *farciminis* within the rat gastrointestinal tract. The authors showed that viable cells were detected in feces and also in the colonic mucosa. However, no direct experimental evidence through an *in situ* characterization was provided and the study was restricted to the colonic region.

The fate of probiotics *in vivo* remains to date poorly understood. In particular, the *in situ* colonization capacity of exogenously supplied probiotics has only been sporadically investigated in the literature and restricted to healthy conditions [26–28]. Most ingested probiotics are probably only transient colonizers of the gut, due to competitive effects exerted by endogenous and well-adapted gut bacteria [57]. In this work, FISH analysis performed with a specific 16S rRNA-targeted probe revealed the presence of *L*. *farciminis* in both ileal and colonic mucosal tissues, despite a different spatial distribution: in the ileum, bacteria were organized in micro-colonies more or less close to the epithelium whereas, in the colon, they were mainly visualized far away from the epithelium. For the latter, as for the endogenous bacteria, the direct contact between the probiotic and the mucosal tissue was likely to be hampered by the inner, firmly attached and dense mucus layer. In contrast, in the small intestine, the discontinuous mucus layer potentially offered more effective interactions with the host [58]. Consistently, the relative abundance of *L*. *farciminis*, as determined by qPCR, was the highest for the ileum. This regio-specificity has previously been reported for lactobacilli [59–60], with preferential colonization sites depending on the species and animal models under study.

To support these findings, *L*. *farciminis* adhesion assays were developed for the intestinal mucosa and purified Muc2. For the two intestinal regions under study, both types of experiments converged on bacterial binding to Muc2 with, from a qualitative point of view, a slightly higher level for the ileum, consistent with the above results. Mucin binding was probably due to the interplay between non-specific physico-chemical interactions (including hydrophobic, electrostatic and van der Waals forces) and specific recognition of bacterial surface components by their mucin O-glycan counterparts, as recently proposed for *Lactococus lactis* and porcine gastric mucin [61–62]. Indeed, for lactic acid bacteria like lactobacilli, a large panel of cell surface proteins, referred to as adhesins, have increasingly been described for their mucus-binding properties, including mucus-binding proteins (MUB) [20], pili [15–16] and multifunctional proteins, such as the elongation factor Tu (EF-Tu) [63], the heat shock protein GroEL [64] and the glyceraldehyde-3-phosphate dehydrogenase (GAPDH) [65]. In line with these results, using whole-genome transcriptome profiling, Marco *et al*. [66] demonstrated that *L*. *plantarum* specifically adapts to the conditions of human and germ-free mice gastrointestinal tracts, notably *via* up-regulation of genes in the cell envelope category, encoding proteinaceous cell surface compounds. For *L*. *farciminis*, the cell surface determinants involved in adhesion and/or muco-adhesion are still largely unknown and will have to be identified in further studies.

A substantial decrease in the *L*. *farciminis* relative abundance was observed for rats submitted to a 4-day WAS, both in the ileal and colonic mucosal tissues. Since no striking differences in binding ability of *L*. *farciminis* to Muc2 were obtained between stressed and sham-stressed animals, despite a modified mucin O-glycosylation pattern [31], one can hypothesize that reduced probiotic levels were rather due to the increase in colonic motility and defecation, induced by WAS in the conditions under study [31]. Nevertheless, we should note that *L*. *farciminis* still exerted its beneficial effects within the rat gastrointestinal tract, notably the strengthening of the mucus and epithelial barriers, impaired by stress [31].

In conclusion, the combination of culture-independent techniques, i.e., FISH and qPCR, allowed demonstrating the presence of exogenous *L*. *farciminis* in the rat gut. Ileum was the primary site compared to colon, as also observed for the SFB population. The presence of the probiotic could be, at least in part, related to binding to the intestinal mucin Muc2, as shown by the coupling of *ex vivo* and *in vitro* approaches. WAS induced a decrease in the *L*. *farciminis* population, even though this lower abundance was not detrimental to maintaining the previously depicted probiotic beneficial effects, such as restoration of the mucus and epithelial barrier function. These data are a major issue concerning the use of probiotics in the management of gut diseases such as IBS, where stress is an associated etiopathogenic and/or aggravating factor.

## Supporting Information

S1 Fig Population of lactobacilli, expressed as their proportion relative to the total bacteria, for vehicle-fed and sham-stressed animals (control group), vehicle-fed and stressed animals (WAS group), *L*. *farciminis*-fed and sham-stressed animals (LF group) and *L*. *farciminis*-fed and stressed animals (LF+WAS group) in the ileum (A) and colon (B). The population of lactobacilli was not statistically different for all conditions under study and reached 50–70% and 20–40% of the total bacteria for the ileum and colon, respectively.
(TIF)
