# Supplementary figures and images for: Spatial Localization and Binding of the Probiotic Lactobacillus farciminis to the Rat Intestinal Mucosa: Influence of Chronic Stress
Source: PLoS One. 2015 Sep 14;10(9):e0136048. doi: 10.1371/journal.pone.0136048 (PMC4569280; doi:10.1371/journal.pone.0136048)

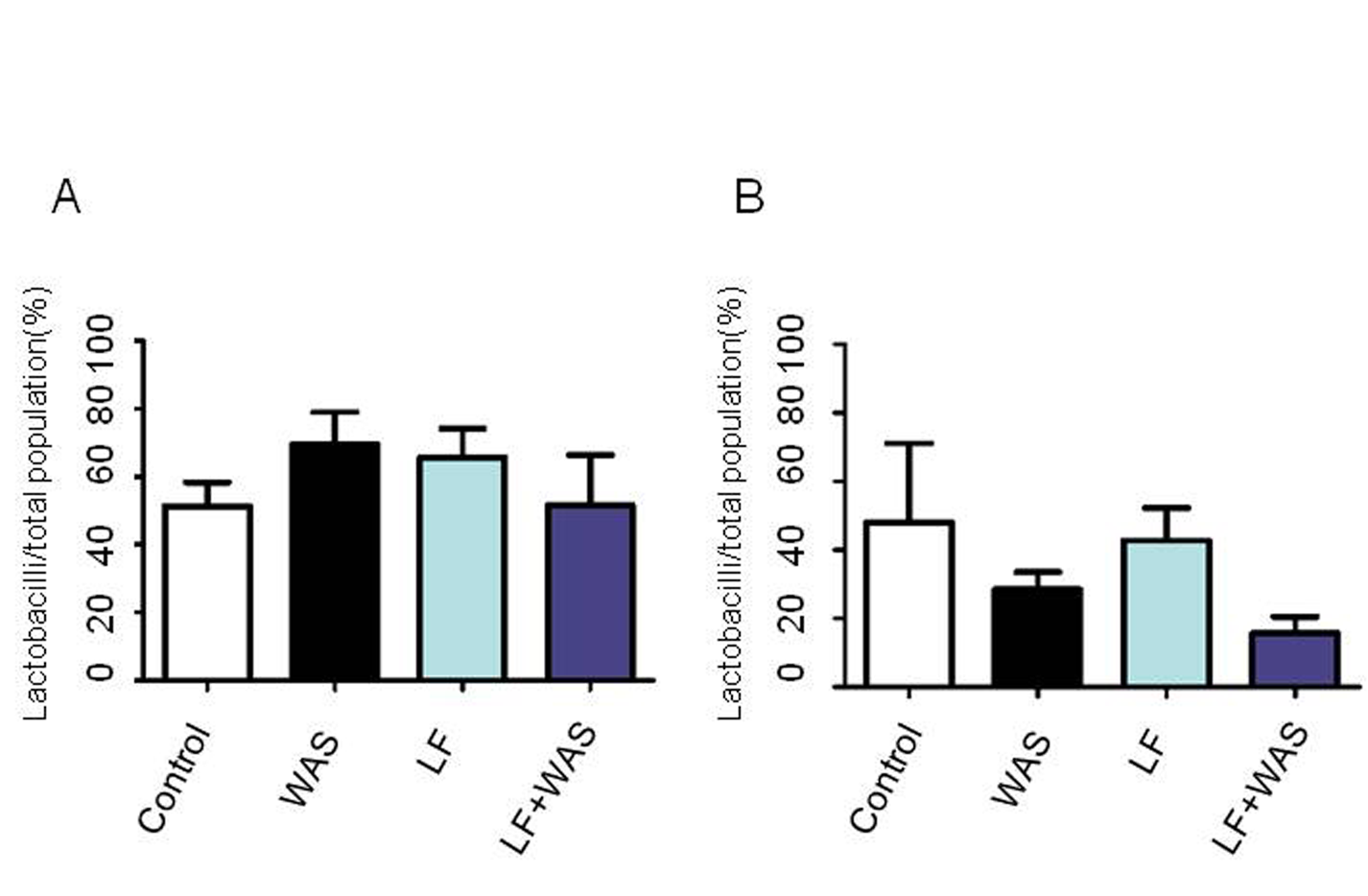

Supplement: S1 Fig — The population of lactobacilli was not statistically different for all conditions under study and reached 50–70% and 20–40% of the total bacteria for the ileum and colon, respectively. (TIF) [file pone.0136048.s001.tif]
